# Extracardiac ^18^F-florbetapir imaging in patients with systemic amyloidosis: more than hearts and minds

**DOI:** 10.1007/s00259-018-3995-2

**Published:** 2018-04-12

**Authors:** T. Wagner, J. Page, M. Burniston, A. Skillen, J. C. Ross, R. Manwani, D. McCool, P. N. Hawkins, Ashutosh D. Wechalekar

**Affiliations:** 1 Department of Nuclear Medicine, Royal Free Hospital, Pond Street, London, UK; 2 National Amyloidosis Centre, University College London (Royal Free Campus), Rowland Hill Street, NW3 2PF, London, UK; 3 Department of Nuclear Medicine, St Bartholomew’s Hospital, Barts Health NHS Trust, West Smithfield, London, UK

**Keywords:** PET, Amyloid, Florbetapir, TTR, AL, Cardiac amyloid

## Abstract

**Purpose:**

^18^F-Florbetapir has been reported to show cardiac uptake in patients with systemic light-chain amyloidosis (AL). This study systematically assessed uptake of ^18^F-florbetapir in patients with proven systemic amyloidosis at sites outside the heart.

**Methods:**

Seventeen patients with proven cardiac amyloidosis underwent ^18^F-florbetapir PET/CT imaging, 15 with AL and 2 with transthyretin amyloidosis (ATTR). Three patients had repeat scans. All patients had protocolized assessment at the UK National Amyloidosis Centre including imaging with ^123^I-serum amyloid P component (SAP). ^18^F-Florbetapir images were assessed for areas of increased tracer accumulation and time–uptake curves in terms of standardized uptake values (SUV_mean_) were produced.

**Results:**

All 17 patients showed ^18^F-florbetapir uptake at one or more extracardiac sites. Uptake was seen in the spleen in 6 patients (35%; 6 of 9, 67%, with splenic involvement on ^123^I-SAP scintigraphy), in the fat in 11 (65%), in the tongue in 8 (47%), in the parotids in 8 (47%), in the masticatory muscles in 7 (41%), in the lungs in 3 (18%), and in the kidney in 2 (12%) on the late half-body images. The ^18^F-florbetapir spleen retention index (SRI) was calculated. SRI >0.045 had 100% sensitivity/sensitivity (in relation to ^123^I-SAP splenic uptake, the current standard) in detecting splenic amyloid on dynamic imaging and a sensitivity of 66.7% and a specificity of 100% on the late half-body images. Intense lung uptake was seen in three patients, one of whom had lung interstitial infiltration suggestive of amyloid deposition on previous high-resolution CT. Repeat imaging showed a stable appearance in all three patients suggesting no early impact of treatment response.

**Conclusion:**

^18^F-Florbetapir PET/CT is a promising tool for the detection of extracardiac sites of amyloid deposition. The combination of uptake in the heart and uptake in the spleen on ^18^F-florbetapir PET/CT, a hallmark of AL, suggests that this tracer holds promise as a screening tool for AL.

**Electronic supplementary material:**

The online version of this article (10.1007/s00259-018-3995-2) contains supplementary material, which is available to authorized users.

## Introduction

Amyloidoses are a group of diseases in which misfolded proteins are deposited in tissues/organs in a highly aggregated form as amyloid fibrils, leading to multisystem disease. About 30 such proteins have been reported to cause human disease, with the two most common types of systemic amyloidosis being transthyretin amyloidosis (ATTR) and acquired monoclonal immunoglobulin light-chain amyloidosis (AL). Age-related ATTR (ATTRwt) occurs due to wild transthyretin protein deposition in the heart, and is an increasingly recognized entity in the elderly. Systemic AL occurs due to an underlying plasma cell dyscrasia secreting an unstable immunoglobulin free light chain. AL is the commonest type of amyloidosis. Appropriate treatment is dependent on accurate identification of the amyloid fibril type and assessment of the disease extent (particularly in the heart) to allow a risk adapted treatment strategy [1, 2].

Imaging of amyloid deposits is challenging and progress has been very slow. Since cardiac involvement is the main cause of morbidity and mortality, focus has been on cardiac imaging. Various modalities including echocardiography (the gold standard for many years), cardiac magnetic resonance imaging (an emerging gold standard) [3, 4], ^99m^Tc-labelled 3,3-diphosphono-1,2-propanodicarboxylic acid (^99m^Tc-DPD) scintigraphy and ^99m^Tc-labelled pyrophosphate (^99m^Tc-PYP) scintigraphy [5] for ATTR cardiac amyloid imaging have become part of routine practice. However, the disease burden in amyloidosis is systemic and imaging other organ systems has not generally been possible. Standard cross-sectional imaging is widely used to document enlargement of an organ, but is not specific for amyloidosis. At our centre (and at the University of Groningen), ^123^I-labelled serum amyloid P component (^123^I-SAP) scintigraphy [6] has been routinely used for visceral amyloid imaging. However, the technical complexity of this procedure has limited its wider use outside the current two centres. ^123^I-SAP scintigraphy is very sensitive and specific for evaluating amyloid in large solid organs [7], but it is not reliable for detecting cardiac involvement. Although SAP uptake is occasionally evident at other sites, the clinical significance of this is not known.

More recently PET tracers used in the diagnosis of Alzheimer’s disease have been used to study cardiac involvement in patients with amyloidosis. The most widely studied tracer is ^11^C-Pittsburgh compound B [8–12], but there is also published literature on the use of ^18^F-florbetapir [13–15] and ^18^F-florbetaben [16]. Despite the different tracers and limited numbers of participants in each of the studies, the technique shows promise and provides good separation between patients confirmed as having cardiac amyloidosis and controls. Although the primary aim of these studies was to study cardiac involvement, several of the papers discussed findings in soft tissue areas outside the heart. Dorbala et al. [13] demonstrated intense ^18^F-florbetapir lung uptake in a patient with clinical manifestations and CT findings of lung amyloid. Osborne et al. [15] generated activity curves over the dome of the liver and found that while the uptake in ATTR patients was similar to that in controls, uptake in patients with AL was significantly increased. Ezawa et al. [12] found many sites of extracardiac uptake and a good clinical correlation between gastric uptake and clinical gastric dysmotillity.

We conducted a study investigating the dynamic uptake of ^18^F-florbetapir centred on the heart and tracer uptake in the remainder of the body 1 h after injection of tracer. This report addresses the uptake of tracer outside the heart.

## Materials and methods

### Recruitment

Patients were recruited for this prospective study of ^18^F-florbetapir between September 2016 and June 2017. The inclusion criteria were as follows:

1. Age ≥40 years
2. Amyloid deposition confirmed on tissue biopsy or imaging
3. Confirmation of amyloid type by either amyloid fibril typing on a tissue biopsy or appropriate genetic testing for mutations (or lack of) in genes for hereditary amyloidosis
4. Evidence of cardiac amyloidosis defined as any one (or more) of the following:
  - Cardiac involvement as defined by the international amyloidosis consensus criteria (including cardiac biomarkers)
  - Cardiac involvement by ^99m^Tc-DPD scintigraphy
  - Cardiac involvement on cardiac MRI imaging
5. Ability to give informed consent

The exclusion criteria were as follows:

1. Inability to lie flat
2. NYHA grade IV heart failure
3. Pregnant or breastfeeding
4. Unwilling to undergo pregnancy test prior to study (in women of child-bearing potential)
5. Known allergy to Amyvid

All patients underwent serial protocolized assessment at the UK National Amyloidosis Centre including full biochemical tests for organ function and serum free light chain measurement, as well as serum and urine protein electrophoresis and immunofixation, cardiac biomarker measurement, echocardiography, cardiac MRI (unless contraindicated), ^123^I-SAP scintigraphy and ^99m^Tc-DPD scintigraphy where indicated. Organ involvement was defined according to international consensus criteria [17].

The study was approved by the institutional research ethics committee and all patients provided written informed consent.

### Image acquisition, reconstruction and analysis

Patients underwent list mode dynamic PET imaging for 60 min using a Siemens Biograph PET/CT scanner following administration of an average of 307 MBq (range 169–370 MBq) of ^18^F-florbetapir. The heart was placed in the centre of the field of view and CT imaging was performed over the same area using automatic exposure control (CARE Dose 4D, CARE kV; Siemens Healthcare) with exposure parameters of 65 mAs and 120 kV. Following dynamic imaging, the patient was asked to void and following this a half-body (skull base to thighs) acquisition was performed (late acquisition) with 3 min per bed position, overlap 42%, and CT with exposure parameters as above. The mean time between injection and the half-body acquisition was 64 min (range 61–69 min).

Images were iteratively reconstructed (two iterations, 21 subsets) using time of flight information and point spread function modelling, with 2 mm gaussian postfiltering The 60 min list mode data were reconstructed into 37 frames (12 frames of 5 s each, 6 frames of 10 s each, 4 frames of 30 s each, 6 frames of 60 s each, 8 frames of 300 s each, and 1 frame of 600 s) replicating the processing method used by Dorbala et al. [13].

^18^F-Florbetapir images were assessed by a nuclear medicine consultant for areas of increased tracer accumulation using a HERMES workstation (HERMES Medical Solutions) and the maximum standardized uptake value (SUVmax) in these areas was assessed using a volume of interest (VOI) of variable size on the late half-body images. Time uptake curves of mean standardized uptake value (SUVmean) against time were produced by manually outlining the left ventricular (LV) myocardium and by placing spherical VOIs on tissues of interest (VOI diameter was varied depending on the size of the organ). VOIs were placed on the dome of the liver, the spleen, the lungs, the bone marrow in the T10 vertebra, the shoulder muscle (infraspinatus or deltoid muscle of the right shoulder) and the axillary fat. Curves were also produced for the stomach lumen and the wall of the stomach when uptake was seen. VOI placement is shown in the figure in the Supplementary material. A spleen retention index (SRI) was calculated as the SUVmean in the spleen VOI from 10 to 30 min divided by the integral of the SUV in a 1 cm diameter spherical VOI placed on the descending aorta from 0 to 20 min after injection.

All available ^123^I-SAP images were scored for uptake in the liver, spleen, kidneys and bone marrow by an experienced amyloid consultant using a four-point scale (0–3) and all ^99m^Tc-DPD images were scored for uptake according to the grading system of Perugini et al. [18]. Normal tracer distributions were inferred from the existing literature [19] and from visual analysis of tracer distribution in our cohort of 17 patients and 20 scans. All scans were first reviewed after completion then reviewed again at the end of the study. Briefly, we considered uptake in the following organs to be physiological on the half-body images obtained at an average of 64 min after injection: low to moderate uptake (SUVmax 4–8) in the liver, low-grade uptake (SUVmax 3–4) in the bone marrow, intense uptake in the small bowel and gallbladder related to physiological excretion of tracer, very low-grade bilateral and symmetrical uptake (SUVmax <3) in the parotids (seen frequently), and low-grade diffuse uptake (SUVmax 3–4) in the tongue (also seen frequently). Uptake at all other sites was presumed to be abnormal and to be related to amyloid deposition.

## Results

Seventeen patients were enrolled in the study, of whom 15 had AL and two had ATTR. Two patients were treatment-naive, one patient was scanned 2 days after the first cycle of chemotherapy and one patient was scanned 9 days after the first cycle of chemotherapy. The patient demographics are summarized in Table 1. Two patients had repeat scans following chemotherapy treatment (one was scanned just under 7 months after completion of chemotherapy and the other was still undergoing chemotherapy but had achieved a complete response). One patient underwent repeat scanning to assess the stability of unexpected lung uptake. All patients showed uptake in the heart as reported previously by others [13–15] and these patients will the subject of a separate article.

**Table 1:** Patient demographics and clinical history

Patient	Age (years)	Sex	Diagnosis
1^a^	80	F	AL, cardiac, renal, liver, splenic involvement
2	46	F	AL, cardiac, renal, splenic involvement
3^a^	61	M	AL, cardiac, soft tissue involvement, mild macroglossia
4	68	M	AL, cardiac, soft tissue involvement, macroglossia
5	50	M	AL, cardiac, liver, splenic, bone marrow involvement, macroglossia, underlying myeloma,
6	51	M	ATTR (wild-type), cardiac involvement
7	73	M	AL, cardiac, renal, splenic involvement, underlying myeloma
8	87	M	ATTR (wild-type), cardiac involvement
9	50	F	AL, cardiac, renal, splenic involvement
10	67	M	AL, cardiac involvement
11	58	M	AL, cardiac and splenic involvement, underlying myeloma
12	47	M	AL, cardiac and renal involvement
13	59	M	AL, cardiac, liver, renal and splenic involvement, underlying myeloma
14^a^	58	M	AL, cardiac, liver renal and splenic involvement
15	46	F	AL, cardiac involvement
16	64	M	AL, cardiac, renal and splenic involvement, submandibular swelling
17	58	M	AL, cardiac and renal involvement, macroglossia, underlying myeloma

^a^Patient underwent a second scan during the study

**Fig. 1:**
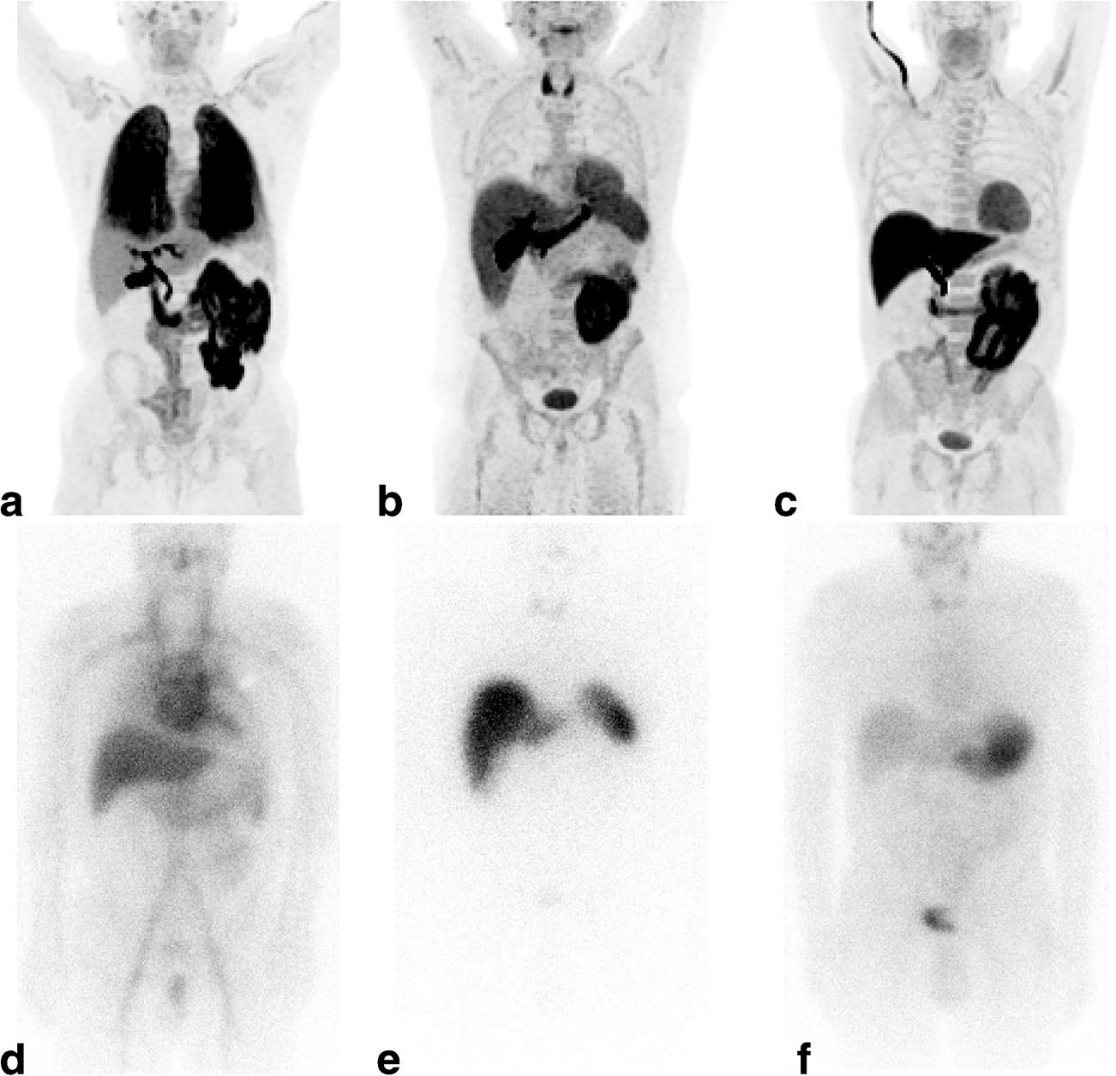
Representative half-body maximum intensity projection ^18^F-florbetapir anterior views (**a–c**) with corresponding ^123^I-SAP planar images for comparison (**d–f**): **a** ^18^F-florbetapir lung uptake (patient 3), **b** ^18^F-florbetapir uptake in the heart, thyroid and spleen (patient 14), **c** ^18^F-florbetapir uptake in the tongue and heart (patient 17), **d** ^123^I-SAP uptake in the liver (patient 3; all other uptake is in the blood pool), **e** ^123^I-SAP uptake in the liver and spleen (patient 14), **f** ^123^I-SAP uptake in the stomach (this is a frequent appearance with ^123^I-labelled tracers and is not an indicator of amyloid deposition). Physiological ^18^F-florbetapir uptake can be seen in the hepatobiliary system of all patients

**Fig. 2:**
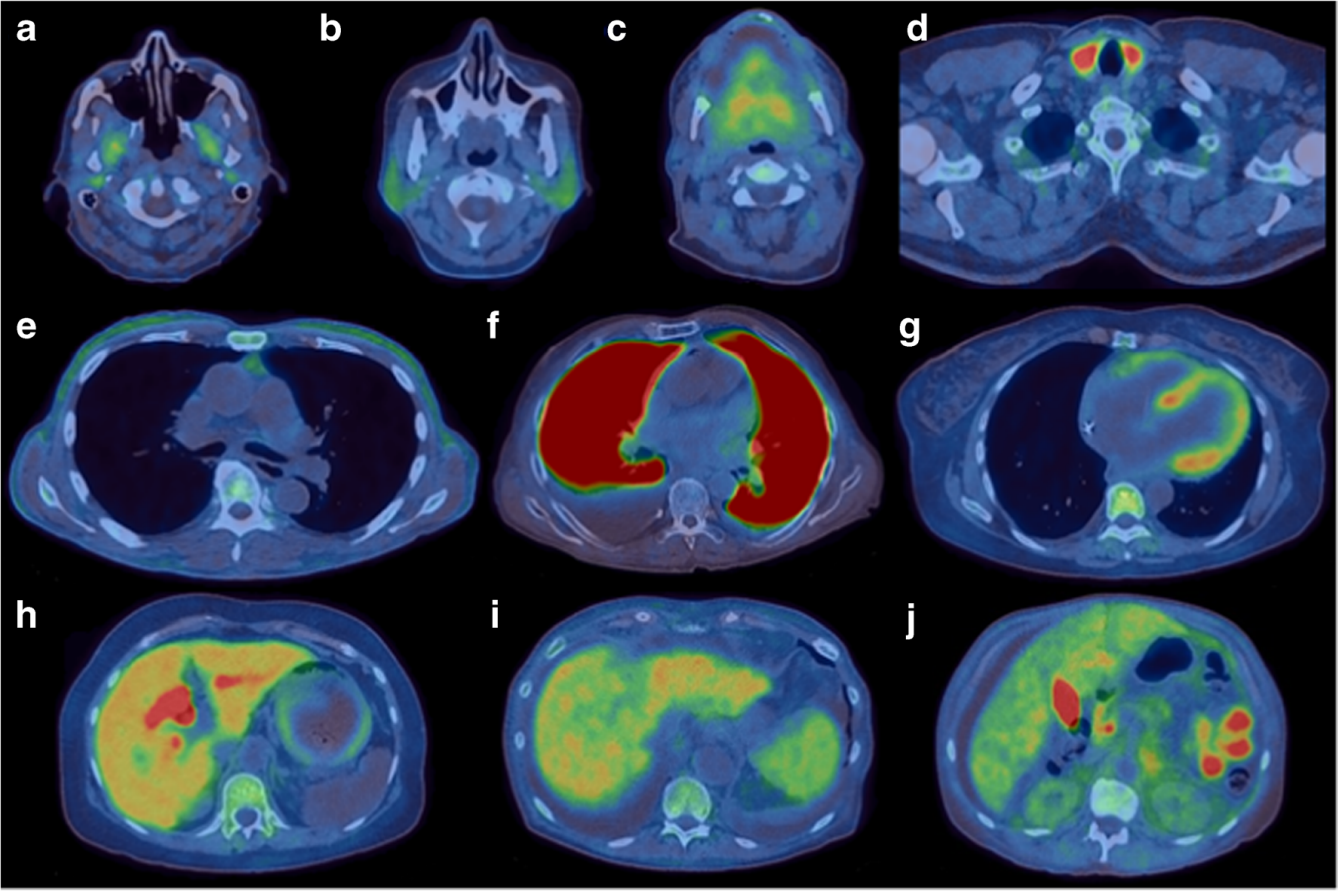
Transaxial ^18^F-florbetapir fused PET/CT images showing representative areas of tracer uptake: **a** pterygoids (patient 3), **b** parotids (patient 2), **c** tongue (patient 17), **d** thyroid (patient 14), **e** subcutaneous and pericardial fat (patient 8), **f** lungs (patient 7), **g** heart and bone marrow (patient 2), **h** liver, bile ducts and stomach wall (patient 2), **I** liver and spleen (patient 1), **j** liver, gallbladder, small bowel and kidneys (patient 13)

All 17 patients showed uptake at one or more extracardiac sites. Representative late half-body images of areas of ^18^F-florbetapir uptake are shown as anterior maximum intensity projections with corresponding ^123^I-SAP images for comparison in Fig. 1 and transaxial fused PET/CT images in Fig. 2. Physiological uptake can be seen in the hepatobiliary system in all patients. Common sites of uptake were fat, parotids, tongue, masticating muscles, spleen and bone marrow. Increased uptake in extracardiac organs seen on the late half-body images is summarized for each patient in Table 2 (along with respective uptake on ^123^I-SAP scintigraphy uptake in liver, spleen, kidneys and bone marrow). Since ^18^F-florbetapir is excreted through the liver, biliary duct and small bowel, uptake in these sites is not included.

**Table 2:** SUV_max_ in extracardiac sites of increased ^18^F-florbetapir uptake on late half-body images and visual scores for previous ^123^I-SAP imaging

Site of uptake		Patient																	Proportion of patients with uptake (%)	Clinical organ involved
		1^a^	2	3^a^	4	5	6	7	8	9	10	11	12	13	14^a^	15	16	17		
Liver	^123^I-SAP score	2	0	0	0	2	0	0	0	0	0	0	0	2	2	0	0	0		
Bone marrow	^123^I-SAP score	1	1	0	0	0	0	0	0	0	0	0	0	1	0	0	0	0		
	^18^F-Florbetapir SUV_max_	4.3 (3.9)	4.8	3.2 (3.5)	4.1	4.2	4.0	1.6	3.7	2.9	6.3	5.5	5.0	4.4	5.2 (6.2)	4.1	3.7	4.6		
Spleen	^123^I-SAP score	2	1	0	0	3	0	2	0	1	0	0	0/1	1	3	0	1	0		
	^18^F-Florbetapir SUV_max_	4.6 (4.9)				4.4		4.9		4.5				4.2	10.2 (9.8)				6/17 (35%)	6/9 (67%)
Kidneys	^123^I-SAP score	0/1	2	0	0	0/1	0	1	0	0	0	0	0	0/1	0/1	0	0	0		
	^18^F-Florbetapir SUV_max_													4.1				2	2/17 (12%)	2/9 (22%)
Fat	^18^F-Florbetapir SUV_max_	3.1 (2.0)			3.8	2.2	2.5		5.3	3.4	1.7	2.4		4.3	3.0 (2.5)	3.1	2.3		12/17(71%)	
Parotids	^18^F-Florbetapir SUV_max_		3.6				3.1				3	3.2	4.2	7.4	5.7 (7.2)	2.2			8/17 (47%)	
Tongue	^18^F-Florbetapir SUV_max_			4.1 (3.9)				3.2		4			3.8	4.3	4.7 (4.9)	2.8		5.0	8/17 (47%)	2/4 (50%)
Submandibular glands	^18^F-Florbetapir SUV_max_		2.4				1.9							3.8	6.0 (5.5)			4.3	5/17 (29%)	
Masticating muscles	^18^F-Florbetapir SUV_max_			4.3 (4.8)				2.7		3.8			2.9		3.6 (3.7)		2.1	3.8	7/17 (29%)	
Larynx/vocal chords	^18^F-Florbetapir SUV_max_			3.1 (2.8)		2.4					3.1								3/17 (18%)	
Lungs	^18^F-Florbetapir SUV_max_			20.2 (16.8)				37		6.8									3/17 (18%)	
Thyroid	^18^F-Florbetapir SUV_max_							4.4						10.0	13.1 (10.3)				3/17 (18%)	
Prostate	^18^F-Florbetapir SUV_max_													4.1	3.2 (3.5)				2/17 (12%)	
Anal canal/sphincter	^18^F-Florbetapir SUV_max_														5.2 (4.8)			3.7	2/17 (12%)	
Thymus/anterior mediastinum	^18^F-Florbetapir SUV_max_		3.2																1/17 (6%)	
Orbital muscles	^18^F-Florbetapir SUV_max_									4.9									1/17 (6%)	
Pancreas	^18^F-Florbetapir SUV_max_									5.6									1/17 (6%)	
Adrenal glands	^18^F-Florbetapir SUV_max_													6.0					1/17 (6%)	
Testicles	^18^F-Florbetapir SUV_max_													3.0					1/17 (6%)	
Clavicle	^18^F-Florbetapir SUV_max_																5.9		1/17 (6%)	
Scalene muscles	^18^F-Florbetapir SUV_max_									4.6									1/17 (6%)	

Blank cells indicate that the site showed no significant uptake on visual assessment

^a^Patient underwent a repeat scan, and the repeat results are shown in parentheses

### Spleen uptake

**Fig. 3:**
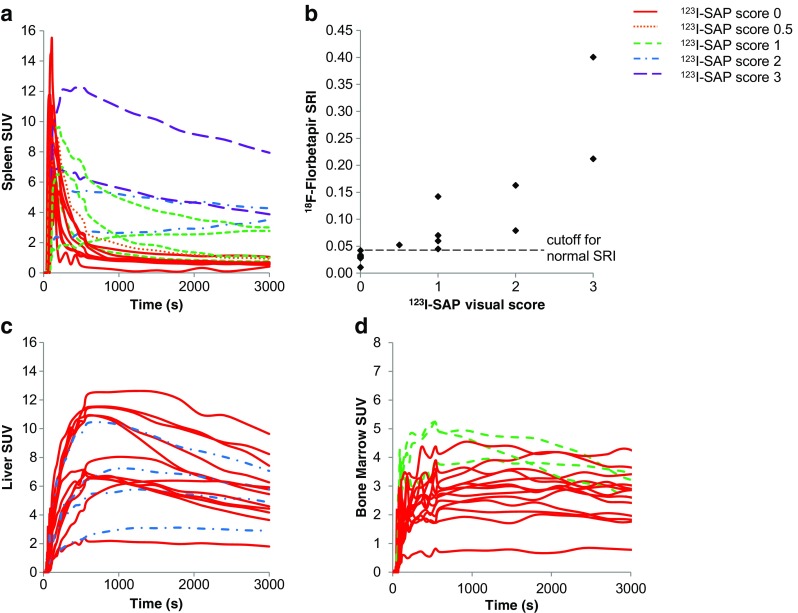
**a** Spleen uptake–time curves for all patients with the ^123^I-SAP spleen scores indicated. **b** ^18^F-Florbetapir spleen retention index (SRI) in relation to ^123^I-SAP spleen score. Patients with a higher ^123^I-SAP spleen score can be seen to have a higher SRI. **c** Liver uptake–time curves for all patients with the ^123^I-SAP liver score indicated (*dashed lines* represent patients with liver uptake on ^123^I-SAP scans). **d** Bone marrow uptake–time curves for all patients with the ^123^I-SAP bone marrow scores indicated (*dashed lines* represent patients with bone marrow uptake on ^123^I-SAP scans)

Spleen uptake (Figs. 1b and 2I) was assessed by splenic retention on dynamic imaging as well as on the late half-body images and compared with spleen uptake using ^123^I-SAP imaging (which is the only imaging modality showing spleen uptake in amyloidosis). In patients who had a negative ^123^I-SAP scan (spleen score 0), the spleen curve peaked sharply during the first 2–3 min, but the SUV fell to background levels by 10–15 min as shown in Fig. 3a. The plot of SRI against ^123^I-SAP visual score is presented in Fig. 3b. All nine patients with splenic uptake on ^123^I-SAP scintigraphy (spleen score >0) had SRIs of >0.045. Using dynamic imaging and a SRI threshold of 0.045, with ^123^I-SAP as the reference, ^18^F-florbetapir had a sensitivity and specificity of 100% for detecting splenic amyloid deposits. However, on the late half-body images spleen uptake was only seen in six of the nine patients with spleen uptake on ^123^I-SAP imaging (Table 2), leading to a specificity of 100% for splenic amyloid identification but a sensitivity of only 66.7% for late half-body imaging with ^18^F-florbetapir. The one third reduction in specificity suggests that the timing of spleen uptake imaging by ^18^F-florbetapir is critical for optimal utility of the scans. Neither of the two patients with ATTR had spleen uptake of ^123^I-SAP or ^18^F-florbetapir.

### Lung uptake

Lung uptake was seen in three patients on the ^18^F-florbetapir half-body images (Table 2, Figs. 1a and 2f). The pattern of uptake was different in all three patients. Patient 3 showed distinct ^18^F-florbetapir uptake with a gradient across the lungs with greater uptake in the posterior than in the anterior aspect (unchanged on repeat imaging 8 months later), patient 7 showed intense and homogeneous uptake, and patient 9 showed heterogeneous focal uptake (corresponding to a lung nodule). The time–activity curves (data not presented) showed that “normal” lung uptake peaked shortly after administration and cleared rapidly, whereas in the three patients with increased lung uptake, the uptake was higher and cleared very slowly. Patient 3 had clinically suspected diffuse pulmonary amyloidosis on high resolution CT scanning with a ground-glass appearance. None of the patients with lung uptake had biopsy-proven lung amyloidosis.

### Liver uptake

^18^F-Florbetapir is excreted via the hepatobiliary system. Liver and bile duct uptake was seen in all patients with the excreted tracer clearing through the small bowel (Fig. 1). Figure 3c shows time–activity curves of SUV_mean_ in VOIs in the liver. There was no significant correlation between ^18^F-florbetapir and ^123^I-SAP uptake, suggesting limited utility of ^18^F-florbetapir for assessing the presence of amyloidosis in the liver.

### Kidney uptake

A total of nine patients had clinical renal involvement (Table 1), six patients had kidney uptake on ^123^I-SAP scintigraphy and only two patients showed low-grade diffuse kidney uptake of ^18^F-florbetapir on the late half-body images (Table 2, Fig. 2j).

### Stomach uptake

^18^F-Florbetapir uptake was seen in the stomach wall (Fig. 2h) and lumen. The uptake seemed to be moving from the stomach wall into the lumen on the dynamic images in 11 patients. On late half-body images, 4 patients (24%) and 12 patients (71%) showed ^18^F-florbetapir uptake in the wall of the stomach and in the lumen, respectively. Uptake in both the wall and lumen was seen in 3 patients (18%). One patient showed different findings on his initial and repeat scans.

### Fat uptake

Some ^18^F-florbetapir uptake was seen in the fat in 12 patients (Table 2, Fig. 2e). including subcutaneous fat (7 patients, 41%), mediastinal fat (4, 24%), and abdominal and pelvic fat (9, 53%). In some patients this uptake was localized to specific regions, while in others the uptake was more widespread.

### Bone marrow uptake

^18^F-Florbetapir uptake was seen in the bone marrow in all patients (Table 2, Figs. 1c and 2g) and some degree of bone marrow uptake is most likely physiological [19]. The mean SUVmax on the half-body images was 4.2 (range 1.6–6.3; Table 2). Three patients had bone marrow uptake on ^123^I-SAP scans (Table 2), and these patients had high early SUVmean values on the ^18^F-florbetapir study. The significance of this uptake is unclear. The bone marrow uptake–time curves are shown in Fig. 3d.

### Tongue uptake

^18^F-Florbetapir uptake was seen in the tongue in eight patients (Table 2, Fig. 2c) with a mean SUVmax of 4.1 (range 2.8–5.0), but of these patients only two had clinically significant macroglossia (Table 1). Two other patients with clinical macroglossia did not show increased ^18^F-florbetapir uptake.

### Other head and neck uptake

Uptake was seen in other tissues in which amyloid deposits are known to form, including the parotid glands (Fig. 2b), thyroid (Fig. 2d) and larynx (Table 2). Most patients had low-grade bilateral and symmetrical parotid uptake considered to be physiological, eight patients had increased parotid uptake with an intensity suggestive of a pathological process with a mean SUVmax of 4.3 (range 2.2–7.4; Table 2). Three patients had increased thyroid uptake (SUVmax 4.4, 10.0 and 31.1; Table 2). Laryngeal uptake was seen in 3 patients (18%; Table 2). Uptake was also seen in the pterygoid (Fig. 2a), orbital and other masticating muscles (Table 2).

### Repeat scans

The three patients with repeat scans showed a stable appearance with all the sites of nonphysiological ^18^F-florbetapir uptake on the baseline half-body images showing similar findings on the repeat half-body images. The uptake–time curves for all organs were unchanged from baseline except the curves for the stomach and liver in patient 14. This may have been physiological or related to diet or fasting, as patients were not required to restrict their eating or drinking prior to the scan.

## Discussion

This is the first study to provide a comprehensive description and analysis of extracardiac sites of ^18^F-florbetapir uptake on PET in patients with proven systemic amyloidosis. The most striking and consistent finding was spleen uptake of ^18^F-florbetapir. This is particularly important, as ^123^I-SAP scintigraphy is the only specific method for spleen imaging and 70% of AL patients have spleen uptake on ^123^I-SAP imaging [6]. Using ^123^I-SAP as the gold standard, on dynamic ^18^F-florbetapir with a SRI cut-off value of 0.045, the sensitivity and specificity were 100%. On late half-body images sensitivity was 66.7% and specificity was 100%. Importantly, there is no clinical splenic involvement in ATTRwt and there was no spleen uptake of ^18^F-florbetapir in the two patients with ATTRwt. The higher sensitivity and specificity of ^18^F-florbetapir on dynamic than on late imaging suggests that earlier imaging (around 15 min after injection) would be best for imaging ^18^F-florbetapir uptake in the spleen. Since splenic involvement is a hallmark of AL, the presence of both cardiac and splenic involvement on ^18^F-florbetapir imaging could potentially be used as an indicator of AL. The combination is generally not seen in other types of amyloidosis except rare hereditary apolipoprotein A1 amyloidosis.

The other striking finding was marked lung uptake in three patients. There is currently no specific imaging technique for detecting lung involvement in patients with systemic AL – diffuse lung involvement remains one of the few areas that are very poorly studied and understood in AL. One of the patients with abnormal lung uptake had interstitial shadowing on high-resolution lung CT images and was suspected to have lung involvement. The significance of lung uptake merits further specific study particularly in patients with proven pulmonary amyloidosis, since a limitation of our study was the lack of histological proof of lung involvement. The altered haemodynamics in cardiac AL also raises the question about poor tracer clearance from the lungs in patients with heart failure since there are no data on this in other settings of heart failure. In their recent study, Ezawa et al. [12] considered ^11^C-PIB lung uptake to be physiological in both patients and controls. The pattern and intensity of uptake in their series was very different from the one in our patient cohort. In our series, uptake was particularly intense and diffuse in two patients and more moderate and heterogeneous in the third patient. None of the other patients demonstrated any lung uptake. The difference in our findings is probably due to the use of different tracers. Further studies investigating the ability of amyloid tracers to detect amyloidosis lung involvement are needed to ascertain their exact role in patient management.

Uptake in fat was identified in more than half the patients which is not dissimilar to the proportion of patients with AL in whom amyloid deposition can be confirmed in abdominal fat aspirates. The uptake of ^18^F-florbetapir in liver was poorly correlated with ^123^I-SAP scintigraphy and there was limited kidney uptake, suggesting limited utility of ^18^F-florbetapir for liver or kidney imaging.

Our results indicate that ^18^F-florbetapir is excreted from the gastric wall into the gastric lumen in most patients and is then cleared into the small bowel but at variable rates (possibly influenced by fasting status and diet). Early gastric uptake and clearance is likely to be physiological since similar stomach activity was also seen in a dosimetry study by Joshi et al. [19]. However, the late stomach wall uptake, noted in 24% of patients, is likely to have been pathological. Diagnosis of gastrointestinal involvement in AL is mainly dependent on symptoms (in the absence of pathology to explain those symptoms). ^18^F-Florbetapir may prove useful for imaging in such patients for diagnosis of gut amyloidosis. Ezawa et al. demonstrated a good correlation between gastric uptake and clinical gastric involvement (decreased gastric motility) in their series [12]. The differences between our findings and those of Ezawa et al. may be due to the different imaging protocols used. We performed a dynamic study over 60 min followed by a half-body acquisition, and Ezawa et al. scanned their patients 30 min after injection. There may also be differences in the results due to the use of different tracers.

Although few patients showed marrow uptake on ^123^I-SAP scintigraphy in the current series, the agreement between the two tracers was striking. Bone marrow uptake of ^18^F-florbetapir occurs in normal controls [19] and hence its utility for bone marrow imaging remains unclear, although patients with higher bone marrow uptake are likely to have pathological bone involvement. Such uptake in a patient with amyloidosis may support a diagnosis of AL as marrow uptake is not seen (on ^123^I-SAP scintigraphy) in any other amyloid type.

Three patients with AL had repeat ^18^F-florbetapir imaging. At the time of their initial imaging, one of these patients was treatment-naive and subsequently went on to have chemotherapy, one was receiving the first cycle of chemotherapy and one had achieved a complete response with chemotherapy. All three patients were in a haematological complete response at the time of repeat imaging. Despite achievement of a haematological response, there were no differences in the distribution and intensity of uptake between initial and repeat imaging. This may have been due to the relatively short time between the baseline and follow-up scans.

Imaging amyloid deposits outside the heart has remained challenging. Recently, most imaging methods such as cardiac MRI and ^99m^Tc-DPD/PYP have focused on cardiac amyloid detection. ^123^I-SAP scintigraphy is used in routine clinical practice at the UK National Amyloidosis Centre for in vivo imaging of amyloid deposits and is the only method that is clinically validated for this purpose. The uptake of ^123^I-SAP correlates with the amount of amyloid [6] with a 90% sensitivity in amyloid A amyloidosis and AL [20]. The main limitation of the technique is that it is available at only two centres worldwide (UK National Amyloidosis Centre and Groningen University) because of its technical complexity. The other limitation is that ^123^I-SAP scintigraphy images the liver, spleen, kidneys and bone, but cannot image the hollow viscera, other soft tissues and heart. Recently, extracardiac uptake of PET tracers (^18^F-florbetapir, ^18^F-florbetaben and ^11^C-PIB) has been described in a few case reports: in the larynx [21], cerebral amyloidoma [22], in the kidneys in ALECT2 type of amyloidosis [23] and in the brain [24]. The only systematic study of extracardiac uptake has recently been published [12]. Half-body images were acquired 30 min after ^11^C-PiB injection and showed abnormal tracer uptake in the spleen, stomach, lachrymal glands, submandibular glands, sublingual glands, lymph nodes, brain, scalp, extraocular muscles, nasal mucosa, pharynx, tongue and nuchal muscles, but most patients were asymptomatic. Ezawa et al. also noted physiological tracer uptake in the urinary tract (kidney, renal pelvis, ureter and bladder) and enterohepatic circulatory system (liver, gallbladder, bile duct and small intestine). Our findings with ^18^F-florbetapir PET imaging are in broad agreement with the results of ^11^C-PiB PET imaging. The main advantage of ^18^F-florbetapir over ^11^C-PiB PET with regard to potential routine clinical implementation is its longer half-life, with no need for an on-site cyclotron and with commercially available solutions for supply and delivery already available in most of Europe and North America.

One of the main limitations of the study was that there was no biopsy proof for every site noted to have abnormal ^18^F-florbetapir uptake. This was not part of the protocol and, due to potential risks, not ethically acceptable in the absence of a specific clinical indication. The other limitation was that this study was limited to cardiac patients (mainly because the study was originally designed to study cardiac amyloidosis) and only patients with AL and ATTRwt were included. Investigation of patients with a wider range of amyloid types and organ involvement is needed to understand physiological and abnormal ^18^F-florbetapir uptake.

In summary, there are a limited number of techniques for imaging extracardiac amyloid deposits. ^123^I-SAP, the gold standard for imaging systemic amyloidosis, has major limitations (it is only available in two centres worldwide, it requires the use of human plasma-derived SAP protein, and the preparation of the tracer is complex). ^18^F-Florbetapir (and other similar tracers) does not have the same limitations and has the great advantage that it is already commercially available worldwide and can be ordered by any appropriately equipped department. Scanning is easy with a half-body scan acquired in less than 30 min as early as 15 min after injection. PET cameras also have better resolution than gamma cameras, along with the possibility of acquiring dynamic scans on cross-sectional images. The combination of heart and spleen uptake of ^18^F-florbetapir may be a specific signature of AL. With its very high cardiac specificity that has already been reported and the additional imaging of extracardiac deposits, ^18^F-florbetapir has the potential to become the main tracer for the diagnosis of systemic amyloidosis. Its role in monitoring disease remains to be clarified, as we did not see any changes on repeat imaging following successful treatment. Large studies in unselected patients with AL are urgently needed to confirm these findings.

## Electronic supplementary material

ESM 1 (DOCX 313 kb)

## References

[1] Wechalekar AD, Gillmore JD, Bird J, Cavenagh J, Hawkins S, Kazmi M (2015). Guidelines on the management of AL amyloidosis. Br J Haematol.

[2] Mahmood S, Palladini G, Sanchorawala V, Wechalekar A (2014). Update on treatment of light chain amyloidosis. Haematologica.

[3] Falk RH, Quarta CC, Dorbala S (2014). How to image cardiac amyloidosis. Circ Cardiovasc Imaging.

[4] Falk RH (2005). Diagnosis and management of the cardiac amyloidoses. Circulation.

[5] Hutt DF, Quigley AM, Page J, Hall ML, Burniston M, Gopaul D (2014). Utility and limitations of 3,3-diphosphono-1,2-propanodicarboxylic acid scintigraphy in systemic amyloidosis. Eur Heart J Cardiovasc Imaging.

[6] Hawkins PN, Lavender JP, Pepys MB (1990). Evaluation of systemic amyloidosis by scintigraphy with 123I-labeled serum amyloid P component. N Engl J Med.

[7] Hawkins PN (2002). Serum amyloid P component scintigraphy for diagnosis and monitoring amyloidosis. Curr Opin Nephrol Hypertens.

[8] Antoni G, Lubberink M, Estrada S, Axelsson J, Carlson K, Lindsjö L (2013). In vivo visualization of amyloid deposits in the heart with 11C-PIB and PET. J Nucl Med.

[9] Lee SP, Lee ES, Choi H, Im HJ, Koh Y, Lee MH (2015). 11C-Pittsburgh B PET imaging in cardiac amyloidosis. JACC Cardiovasc Imaging.

[10] Pilebro B, Arvidsson S, Lindqvist P, Sundström T, Westermark P, Antoni G (2018). Positron emission tomography (PET) utilizing Pittsburgh compound B (PIB) for detection of amyloid heart deposits in hereditary transthyretin amyloidosis (ATTR). J Nucl Cardiol.

[11] Kero T, Lindsjö L, Sörensen J, Lubberink M (2016). Accurate analysis and visualization of cardiac 11C-PIB uptake in amyloidosis with semiautomatic software. J Nucl Cardiol.

[12] Ezawa N, Katoh N, Oguchi K, Yoshinaga T, Yazaki M, Sekijima Y (2018). Visualization of multiple organ amyloid involvement in systemic amyloidosis using 11C-PiB PET imaging. Eur J Nucl Med Mol Imaging.

[13] Dorbala S, Vangala D, Semer J, Strader C, Bruyere JR, Di Carli MF (2014). Imaging cardiac amyloidosis: a pilot study using 18F-florbetapir positron emission tomography. Eur J Nucl Med Mol Imaging.

[14] Wells K, Osborne D, Stuckey A, Wilson S, Wall J, Solomon A (2013). 18F Florbetapir PET/CT cardiac amyloid imaging in patients with systemic amyloidosis. J Nucl Med.

[15] Osborne DR, Acuff SN, Stuckey A, Wall JS (2015). A routine PET/CT protocol with streamlined calculations for assessing cardiac amyloidosis using 18F-florbetapir. Front Cardiovascular Med..

[16] Law WP, Wang WY, Moore PT, Mollee PN, Ng AC (2016). Cardiac amyloid imaging with 18F-florbetaben PET: a pilot study. J Nucl Med.

[17] Gertz MA, Comenzo R, Falk RH, Fermand JP, Hazenberg BP, Hawkins PN (2005). Definition of organ involvement and treatment response in immunoglobulin light chain amyloidosis (AL): a consensus opinion from the 10th International Symposium on Amyloid and Amyloidosis, Tours, France, 18-22 April 2004. Am J Hematol.

[18] Perugini E, Guidalotti PL, Salvi F, Cooke RMT, Pettinato C, Riva L (2005). Noninvasive etiologic diagnosis of cardiac amyloidosis using 99mTc-3,3-diphosphono-1,2-propanodicarboxylic acid scintigraphy. J Am Coll Cardiol.

[19] Joshi AD, Pontecorvo MJ, Adler L, Stabin MG, Skovronsky DM, Carpenter AP (2014). Radiation dosimetry of florbetapir F 18. EJNMMI Res.

[20] Hazenberg BP, van Rijswijk MH, Piers DA, Lub-de Hooge MN, Vellenga E, Haagsma EB (2006). Diagnostic performance of 123I-labeled serum amyloid P component scintigraphy in patients with amyloidosis. Am J Med.

[21] García-González P, Sánchez-Jurado R, Cozar-Santiago MP, Ferrando-Beltrán M, Pérez-Rodriguez PL, Ferrer-Rebolleda J (2017). Laryngeal and cardiac amyloidosis diagnosed by 18F-florbetapir PET/CT. Rev Esp Med Nucl Imagen Mol.

[22] Villarejo-Galende A, Sarandeses P, Penas-Prado M, Hernández-Laín A, Ramos A, Hernández-Martínez AC (2015). PET-florbetapir findings in primary cerebral amyloidoma. J Neurol.

[23] Leung N, Ramirez-Alvarado M, Nasr SH, Kemp BJ, Johnson GB (2017). Detection of ALECT2 amyloidosis by positron emission tomography-computed tomography imaging with florbetapir. Br J Haematol.

[24] Genovesi D, Vergaro G, Emdin M, Giorgetti A, Marzullo P (2017). PET-CT evaluation of amyloid systemic involvement with [18F]-florbetaben in patient with proved cardiac amyloidosis: a case report. J Nucl Cardiol.

