# Supplementary figures and images for: Extracardiac 18F-florbetapir imaging in patients with systemic amyloidosis: more than hearts and minds
Source: Eur J Nucl Med Mol Imaging. 2018 Apr 12;45(7):1129–38. doi: 10.1007/s00259-018-3995-2 (PMC5953997; doi:10.1007/s00259-018-3995-2)

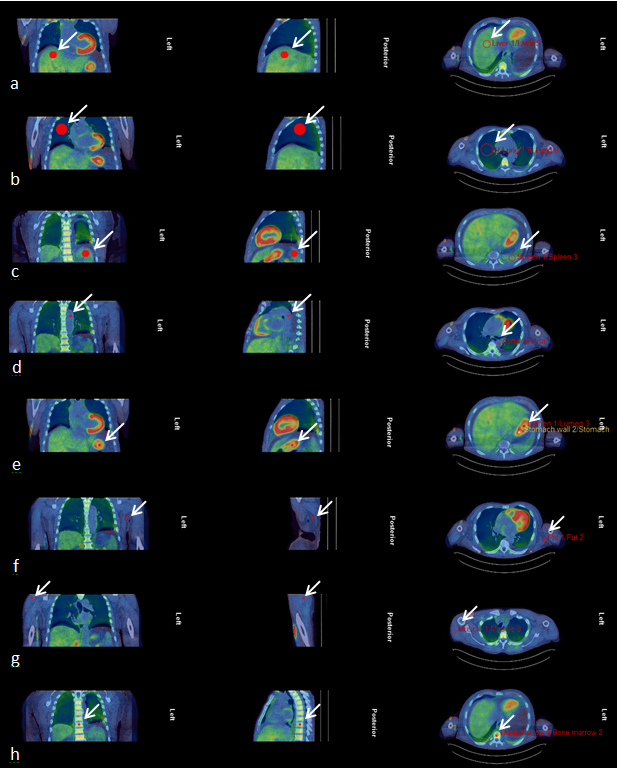

Supplement: Supplementary file 1 — (DOCX 313 kb) [file 259_2018_3995_MOESM1_ESM.docx]
